# Identification of SARS-CoV-2-specific cytotoxic lymphocytes in cerebrospinal fluid of a patient with cerebral demyelination Post mRNA vaccination

**DOI:** 10.3389/fimmu.2026.1920061

**Published:** 2026-09-21

**Authors:** Takaya Kitano, Masakazu Ishikawa, Makoto Kinoshita, Keigo Kihara, Shuhei Sakakibara, Tomohiro Yata, Ryohei Yamamura, Hideki Mochizuki, Masaru Yokoe, Tatsusada Okuno, Daisuke Okuzaki

**Affiliations:** 1 Department of Neurology, Toyonaka Municipal Hospital, Osaka, Japan; 2 Department of Neurology, Graduate School of Medicine, the University of Osaka, Osaka, Japan; 3 Laboratory of Human Immunology (Single Cell Genomics), WPI Immunology Frontier Research Center, the University of Osaka, Osaka, Japan; 4 Center for Infectious Disease Education and Research, the University of Osaka, Osaka, Japan; 5 Laboratory of Immune Regulation, Immunology Frontier Research Center, the University of Osaka, Osaka, Japan; 6 Graduate School of Medical Safety Management, Jikei University of Health Care Sciences, Osaka, Japan; 7 National Hospital Organization Osaka Toneyama Medical Center, Osaka, Japan; 8 Genome Information Research Center, Research Institute for Microbial Diseases, the University of Osaka, Osaka, Japan; 9 Institute for Open and Transdisciplinary Research Initiatives, the University of Osaka, Osaka, Japan

**Keywords:** demyelination, multiple sclerosis, natural killer cells, repertoire, single cell, T cell, vaccination

## Abstract

**Introduction:**

Some cerebral demyelinating lesions occur after vaccination. We aimed to elucidate the precise roles of vaccine-activated immune cells and antigen-specific lymphocytes in the central nervous system of a patient who developed cerebral demyelination after vaccination.

**Methods:**

Using comprehensive single-cell RNA sequencing and immune repertoire analysis combined with a lymphocyte stimulation assay, we characterized the immunological landscape of a patient who developed right-hand clumsiness after receiving an mRNA vaccine against SARS-CoV-2 and was subsequently diagnosed with tumefactive cerebral demyelination.

**Results:**

Natural killer cells and effector CD8+ T cells in the cerebrospinal fluid were more cytotoxic and activated than those in healthy controls. Effector CD8+ T cells specific to SARS-CoV-2 spike peptides were identified in the cerebrospinal fluid. The proportion of these cells in peripheral blood decreased after corticosteroid therapy, in parallel with clinical improvement and a reduction in lesion size.

**Discussion:**

Although a direct causal relationship between vaccination and demyelination remains uncertain, our findings suggest that vaccine-activated cytotoxic lymphocytes contribute to immune-mediated demyelinating events in the central nervous system.

## Introduction

Central nervous system (CNS) demyelinating events have been reported following SARS-CoV-2 vaccination, although findings have varied across studies (1–10). Neurological manifestations associated with SARS-CoV-2 infection are thought to involve immune-mediated mechanisms, including excessive myelin phagocytosis caused by microglial dysfunction, rather than direct viral invasion of the CNS (11–13). However, the immune landscape underlying post-vaccination demyelination remains largely unexplored. In a series of 19 patients who developed CNS immune-mediated disorders following SARS-CoV-2 vaccination, known neural autoantibodies were rarely detected (14). In particular, it remains unknown whether antigen-specific lymphocytes activated in the periphery after vaccination subsequently enter the CNS.

To characterize the immune landscape of post-vaccination demyelination and to determine whether peripherally activated antigen-specific lymphocytes enter the CNS, we investigated a patient with recurrent tumefactive cerebral demyelination following BNT162b2 vaccination. We performed comprehensive single-cell RNA sequencing (scRNA-seq) and immune repertoire analyses of cerebrospinal fluid (CSF) and peripheral blood, combined with a lymphocyte stimulation assay to determine antigen specificity.

## Case

A 66-year-old male patient was admitted because of dysarthria and clumsiness of the right hand that began 2 weeks after the first dose of BNT162b2. He had been diagnosed with a subcortical lesion in the left frontoparietal lobe 10 years before this admission (**Figure 1A**). At that time, his symptoms improved and the lesion diminished spontaneously (**Figure 1B**), but he was lost to follow-up. On admission, a neurological examination revealed paresis in his right face and hand. Brain MRI showed a new lesion (**Figure 1C**). His serum was negative for known antibodies (**Supplementary Table 1**). CSF cell count was 3 cells/μL. The protein level was slightly elevated, and the myelin basic protein level was elevated to 1800 pg/mL. He was diagnosed with tumefactive demyelination. The symptoms resolved, and the lesion shrank after prednisone therapy (**Figure 1D**; **Supplementary Figure 1**).

**Figure 1:**
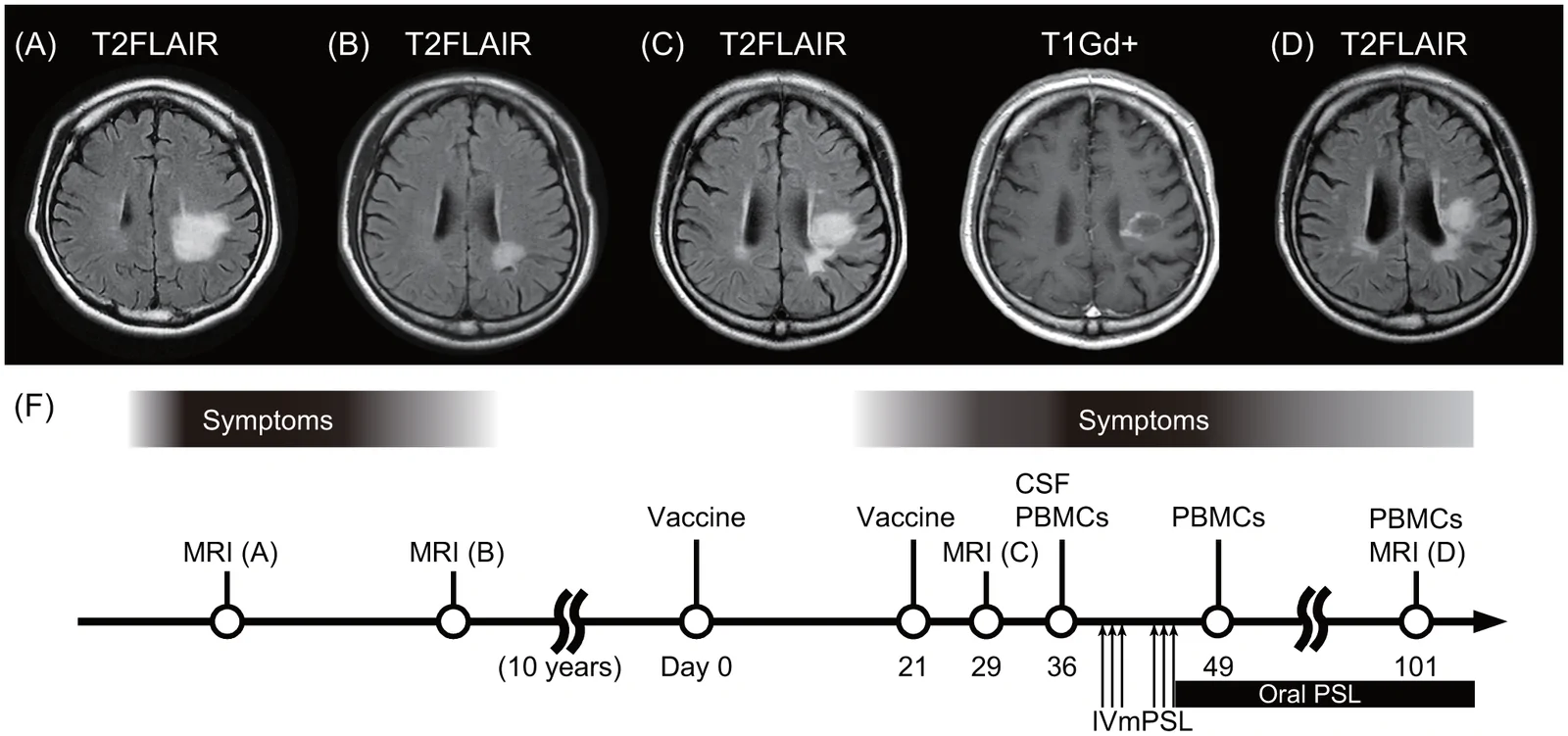
MRI images and summary of the case. **(A, B)** Axial fluid-attenuated inversion recovery (FLAIR) images on the previous admission. **(A)** when the patient developed symptoms; **(B)** after the spontaneous resolution. **(C, D)** On the present admission after the vaccination. **(C)** FLAIR image and contrast-enhanced T1-weighted image that shows the open-ring enhancement; **(D)** FLAIR image after the steroid therapy. **(E)** Summary of the course.

## Methods

### Sample preparation

CSF cells and peripheral blood mononuclear cells (PBMCs) were subjected to scRNA-seq and TCR-seq. CSF during the acute phase of the disease was obtained from the patient before steroid therapy initiation. Peripheral blood was obtained on the same day as the CSF, at 2 weeks and 3 months after the steroid therapy initiation. Peripheral blood samples were obtained in heparin tubes, and PBMCs were separated using Ficoll-Paque PLUS (GE Healthcare, Chicago, IL, USA). Obtained CSF cells and PBMCs were resuspended in CELLBANKER (Amsbio, Cambridge, MA, USA) and stored at -80 °C until analysis. Written informed consent was obtained from the participant, and the protocol was reviewed and approved by the Ethics Committees of the Toyonaka Municipal Hospital (approval #2021-05-02) and the University of Osaka Hospital (approval #22463) in accordance with the Declaration of Helsinki before sample collection.

### Single-cell sequencing and bioinformatic analysis

Cell preparation for single-cell sequencing followed our previously published paper (15). Gene expression, T cell receptor (TCR), and feature barcode libraries were sequenced using the DNBSEQ-G400 (MGI) to achieve a minimum of 20,000 paired-end reads per cell for gene expression and 5,000 paired-end reads per cell for TCR and feature barcode libraries. Sequencing data obtained from MGISEQ-G400 were aligned to the GRCh38 genome using Cell Ranger (v.6.1.0). A clonotype was considered expanded when >1 cells with that clonotype exist in the CSF. We also obtained the public data deposited by Gate et al. as a healthy control (16). These data included CSF cells obtained from eight individuals selected from a cohort of 34 participants (mean age, 69 years), with selection balanced for sex and age. These healthy individuals had not been vaccinated against SARS-CoV-2, had no acute infectious diseases, and were in good physical health. Filtered matrices produced by Cell Ranger were loaded into the R package Seurat (v.4.0), and data filtering, normalization, scaling, dimensional reduction, clustering, and visualization were conducted using Seurat (17). Normalization and batch correction between the publicly available data and our own dataset were conducted via Seurat’s SCTransform function. Cell-type identification was performed manually for CSF cells and automatically using ScType for PBMCs (18). Genes with log2FC > 0.25 and FDR < 0.05 were defined as upregulated using the FindMarkers function. Gene Ontology (GO) enrichment analysis was conducted using the compareCluster function in the R package clusterProfiler (19). Cell-cell interaction was analyzed by CellPhoneDB (v5) using default parameters (20). The number of inferred significant interactions was calculated for each source-target pair, and the increase in the patient compared to healthy controls was visualized using network plots. The pairs and interaction scores of ligands and receptors related to chemokine and cytokine (Signaling by Interferon/Interleukin/Tumor necrosis factor) were visualized using the circlize package.

### PBMC stimulation by SARS-CoV-2 peptide

PBMCs collected before steroid administration were cultured for 24 h at 37 °C in a humidified incubator with 5% CO_2_. The sample was divided into two groups, one stimulated with SARS-CoV-2 Spike peptide and the other with influenza A NP peptide. In the SARS-CoV-2 spike peptide stimulation group, we stimulated samples with 1 μg/ml S-peptide pool: PepMixTM SARS-CoV-2 (Spike Glycoprotein) (PM-WCPV-S-1, JPT Peptide Technologies, Berlin, Germany) or 0.4% DMSO as a negative control for 24 h at 37 °C in a humidified incubator with 5% CO_2_. For the influenza peptide stimulation group, we stimulated samples with 1μg/ml PepTivatorInfluenza A (H1N1) NP (Miltenyi Biotec, Bergisch Gladbach, Germany) or cell culture as a negative control for the same duration. After stimulation, cell surface marker staining was performed.

### Flow cytometry and bulk TCR sequencing of antigen-specific T cells

PBMCs were incubated with FcR Blocking Reagent (Miltenyi Biotec, Bergisch Gladbach, Germany) and stained for cell surface markers. Lymphocyte subsets were identified using a combination of the following fluorescent-labeled antibodies: CD3-PE, CD8-BV510 (BioLegend, San Diego, CA, USA); CD4-BV605, CD14-PEcy7, CD19- PEcy7 (BD Biosciences, San Jose, CA, USA). Cells responsive to the stimulation were defined as double positivity for CD137-APC and CD69-FITC (BioLegend, San Diego, CA, USA) for CD8+ T cells, and double positivity for OX-40-FITC and 4-1BB-APC for CD4+ T cells. The flow cytometry results were analyzed using FACS Aria II (BD Biosciences, San Jose, CA, USA) and FlowJo v.10.8 software (BD Biosciences, San Jose, CA, USA).

After division into responsive and non-responsive groups, cells were directly sorted into Qiazol (Qiagen, Venlo, Netherlands). RNA from sorted cells was extracted according to the manufacturer’s protocol. TCR libraries were prepared by using SMARTer Human TCR a/b Profiling Kit (Takara Bio Inc., Shiga, Japan) according to the manufacturer’s protocol (Takara Bio Inc.). The generated libraries were measured for their DNA concentration by Qubit and assessed for their size by an Agilent 2200 TapeStation system (Agilent Technologies, Santa Clara, CA, USA). TCR sequencing was performed on an Illumina MiSeq sequencer using the 600-cycle MiSeq reagent kit v3 (Illumina) with pair-end, 2 × 300 base pair reads. VDJ alignment and clonotyping of the generated raw reads were processed using the MiXCR software package (v.3.0.13) (21).

### KIR genotyping

KIR genotyping was performed using a hybridization capture–based next-generation sequencing method by a commercial provider (GenoDive Pharma Inc., Japan). Library preparation was conducted using the KAPA HyperPlus kit, followed by targeted enrichment of KIR genes using custom-designed biotinylated oligonucleotide probes. The enriched libraries were sequenced on an Illumina NextSeq 2000 platform. The sequencing of 16 KIR genes (KIR2DL1, KIR2DL2, KIR2DL3, KIR2DL4, KIR2DL5, KIR2DP1, KIR2DS1, KIR2DS2, KIR2DS3, KIR2DS4, KIR2DS5, KIR3DL1, KIR3DL2, KIR3DL3, KIR3DP1, and KIR3DS1) was performed. Sequence data were analyzed using NGSengine software (GenDX) to assign KIR alleles based on comparison with a reference database, enabling high-resolution allele typing. Copy number variation of each KIR gene was estimated using the DepthOfCoverage tool in the Genome Analysis Toolkit. Read depth data were analyzed to infer gene copy number relative to the expected genomic baseline. Based on combined allele typing and copy number estimation, KIR haplotypes were determined.

### Data and code availability

The sequencing data generated and analyzed in this study are available in the Gene Expression Omnibus under accession number GSE296814. All analysis scripts and code required to reproduce the main analyses are publicly available at https://github.com/amufaamo/SARS-CoV2_CSF_CD8_Tcells.

## Results

### scRNA-seq analysis

scRNA-seq analysis of CSF cells (**Figure 2A**) identified eight cell types based on marker gene expression (**Figures 2B, C**). The proportions of NK cells (11.3% vs. 2.0%), B cells (2.2% vs. 0.3%), monocytes (8.0% vs. 1.1%), and myeloid dendritic cells (MDCs) (10.1% vs. 2.6%) were higher in the patient’s CSF than in controls (**Figure 2D**). The number of each cell type is shown in **Supplementary Table 2**. When all cells were analyzed together without stratification by cell type, differential expression analysis identified 749 upregulated genes and 81 downregulated genes in the patient sample compared with the control samples. Most enriched terms in the upregulated genes were related to T cell and lymphocyte activation (**Figure 2E**). Next, gene expression was compared between the patient and the controls within each cell type. The number of differentially expressed genes by cell type is shown in **Supplementary Figure 2**. These differentially expressed genes were then subjected to enrichment analysis. The upregulated pathways were exclusive to effector CD8+ T cells (CD8T_eff_), effector CD4+ T cells (CD4T_eff_), naive CD4+ T cells, and NK cells (**Figure 2F**). Clustering of the upregulated pathways revealed that those related to lymphocyte activation were enriched in all four cell types (**Figure 2G**). Pathways related to leukocyte-mediated cytotoxicity were enriched in CD8T_eff_ and NK cells. Upregulated genes in the patient’s CSF included *NKG7*, *PRF1*, and *KLRK1* (CD8T_eff_) and *NKG7*, *PRF1*, *GZMB*, and *FCGR3A* (NK) (**Figure 2H**). A cluster of mature NK cells with elevated cytotoxicity was expanded in the patient’s CSF (**Supplementary Figure 3**). These results suggest that lymphocytes are activated and more cytotoxic in the patient’s CSF.

**Figure 2:**
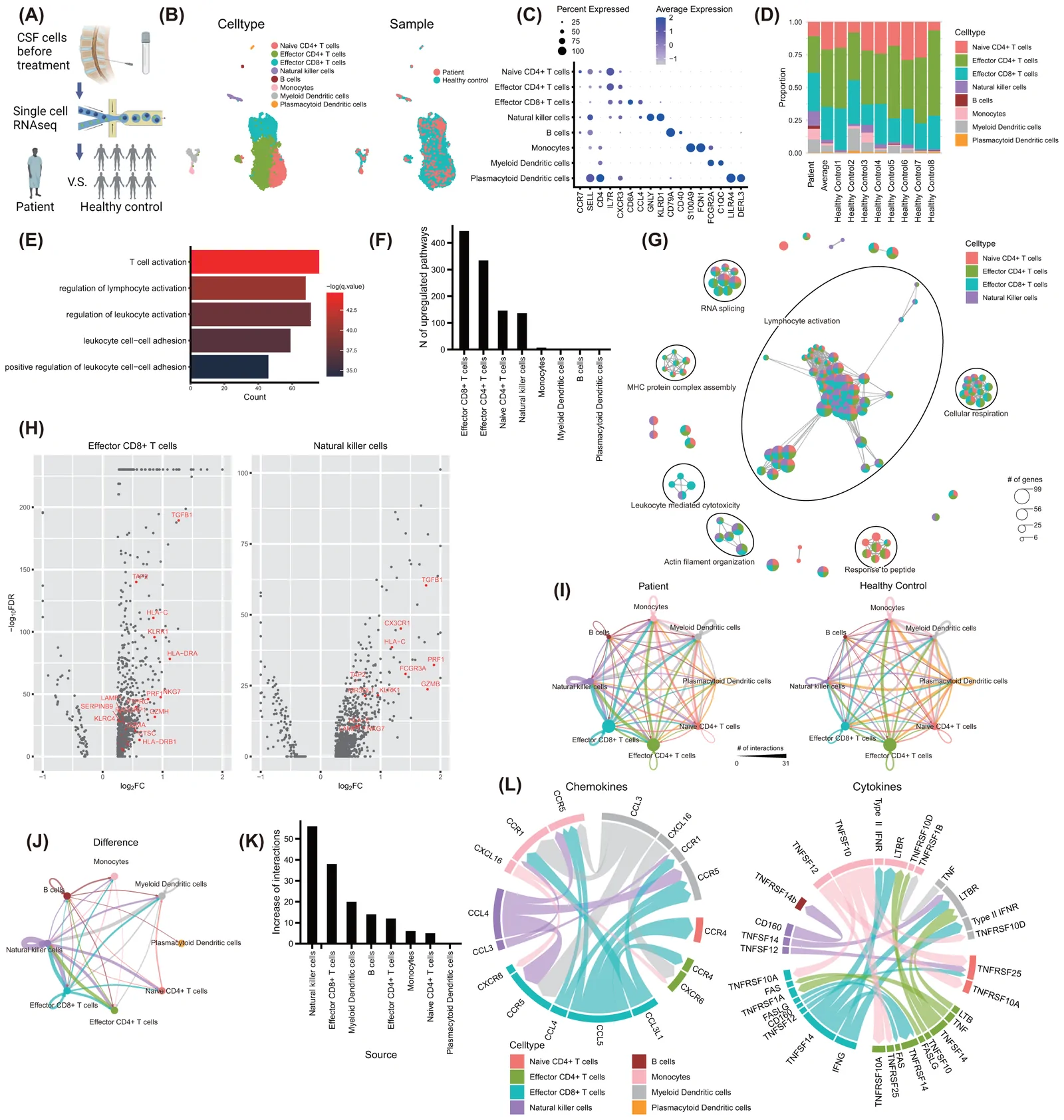
Single-cell RNA-sequencing showed effector CD8 T cell activation. **(A)** Schematic summary of the analysis. **(B)** UMAP plot for 495 cells from this patient before treatment and 9,756 cells from eight healthy individuals. **(C)** Dot plots for the marker gene expression. **(D)** The proportion of each cell type in the patient and healthy individuals. **(E)** Enrichment analysis comparing the patient’s CSF with healthy controls. The top 5 pathways with the lowest FDR are visualized. **(F)** Enrichment analysis using the upregulated genes in the patient was performed according to cell type. The number of enriched pathways in each cell type (GO, Biological Process) is shown. **(G)** Top 50 significantly enriched pathways in naïve CD4+ T cells, CD4T_eff_, CD8T_eff_, and NK are visualized as a network plot. Each node represents a pathway, node size represents the number of genes included in the pathway, and edges represent the similarity of the connected nodes. Fill colors represent the cell type in which the pathway was found to be enriched. **(H)** In NK cells and CD8T_eff_, gene expression in the patient’s CSF was compared with that in healthy controls. Upregulated genes related to cytotoxicity in the patient’s CSF cells are highlighted in red. The values of -log (adjusted p value) and log_2_(FC) are truncated for visualization. **(I, J)** Network plots of the cell–cell interaction. **(I)** in the patient’s CSF (left) and healthy controls’ (right). Edge width represents the number of inferred interactions, and node size represents the number of cells. **(J)** edge width and the number on it represent the difference (increase only) in the number of inferred interactions in the patient’s CSF compared with healthy controls. **(K)** Sum of the increase in the interactions according to the cell type. **(L)** Chord diagrams of the inferred ligand–receptor pairs involved in chemokine and cytokine signaling. The pairs found only in the patient are visualized, and the width of the chord represents interaction scores.

Cell–cell interaction analysis revealed that the number of inferred interactions was elevated in the patient, especially those originating from NK cells and CD8T_eff_ (**Figures 2I–K**). In the patient, signaling from NK cells and CD8T_eff_ to the other leukocytes through chemokines, such as CCL3, CCL4, and CCL5, was inferred, as well as MDC stimulation by NK cells through *TNFSF1*4-*LTBR* signaling and CD8T_eff_ through interferons (**Figure 2L**). Our data suggest that activated lymphocytes with enhanced cytotoxicity and communication between MDCs were distinctive features of CSF cells derived from the patient.

### Repertoire analysis of T cells and their responsiveness to SARS-CoV-2 spike peptide

To explore the role of antigen-specific immunity, we examined the clonal expansion of T cells and how much the expanded clonotypes were shared between CSF and peripheral blood using single-cell T cell receptor (TCR) CDR3 sequencing in the patient’s CSF and 23,940 peripheral blood mononuclear cells (PBMCs) (**Figure 3A**). PBMCs were annotated automatically (**Supplementary Figure 4**). TCR repertoire analysis revealed clonal expansion in the CSF T cell population, in particular CD8T_eff_, indicating an antigen-specific T cell response. We found 99 clonotypes of CD8T_eff_, 18 of which were expanded, and 116 clonotypes of CD4T_eff_, 10 of which were expanded. Cells whose CDR3 sequence was shared between CSF were mainly identified in CD8T_eff_ (**Figure 3B**). Among the expanded CD8T_eff_ in the patient’s CSF, 11 were shared with peripheral blood, while only 1 was shared with peripheral blood in CD4T_eff_ (**Figure 3C**). These data suggest that antigen-specific CD8T_eff_ moved from the periphery to the CNS.

**Figure 3:**
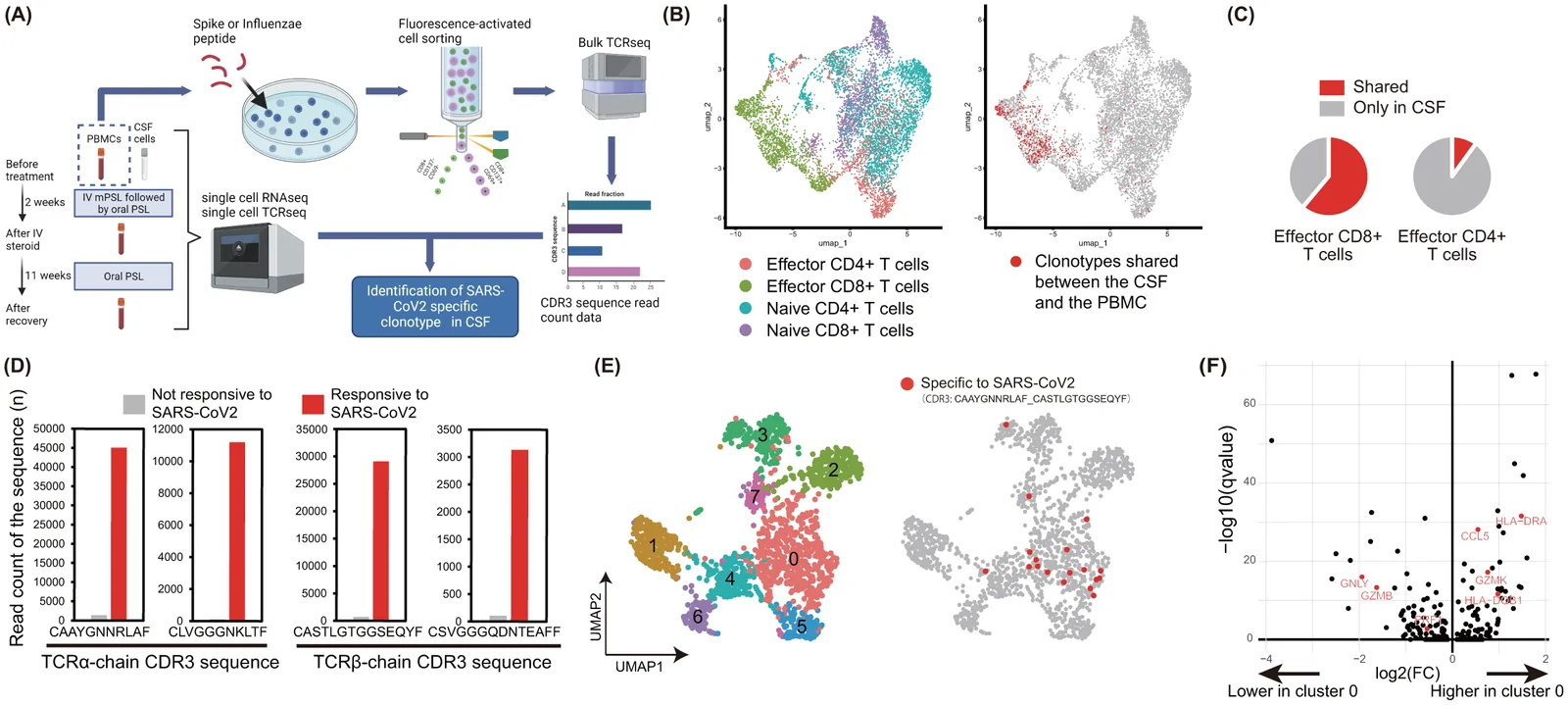
TCR sequencing revealed that effector CD8+ T cells specific to SARS-CoV-2 were expanded in the patient’s CSF. **(A)** Schematic summary of the experiments. **(B)** UMAP plot for 7,650 peripheral T cells from the patient. Cells with a clonotype that exists in both CSF and peripheral T cells are highlighted. **(C)** Proportion of the clonotypes shared between CSF and PBMCs among the expanded clonotypes in the CSF according to cell type. A clonotype was judged as expanded when two or more cells with the same CDR3 sequence were identified in the sample. **(D)** Two clonotypes were identified as responsive to SARS-CoV-2 peptide because their TCRα- and TCRβ-chain CDR3 sequences were mostly detected among CD137+CD69+ cells after the stimulation. Read count of each CDR3 sequence according to the expression of CD137 and CD69 is presented. **(E)** UMAP plot for 1,612 CD8T_eff_ in the peripheral blood. Left, unbiased clustering; right, cells specific to SARS-CoV-2. **(F)** Comparison of the expression of the selected genes in the patient’s CD8T_eff_ between cluster 0 and the others.

To examine the antigen specificity of the T cells, we searched for SARS-CoV-2 S protein-specific TCR clonotypes in PBMCs from the same patient and examined their overlap with TCRs derived from the CSF T cells. We stimulated the patient’s PBMCs with a pool of SARS-CoV-2 spike- or influenza NP-derived peptides (**Figure 3A**). In response to the S protein and influenza NP peptide stimulation, 0.46% and 0.60% of CD8+ T cells, respectively, were activated (CD137+ and CD69+). In contrast, only 0.15% of CD8+ T cells were activated following stimulation with vehicle alone (**Supplementary Figure 5**). We subsequently analyzed the CDR3 amino acid sequence by utilizing bulk TCR sequencing of the sorted cells according to the response to the stimuli. The frequency distributions of clonotypes defined by CDR3 differed between the activated and non-activated fractions. Four and three TCRβ clonotypes in the SARS-CoV-2 and influenza peptide-activated cells, respectively, were expanded, and each accounted for > 5% of total reads, whereas no clonotypes in the non-activated fraction reached 2% (**Supplementary Figure 6**). One expanded clone of CD8T_eff_ in the CSF possessed a TCR repertoire composed of TCRα with a CDR3 sequence, CAAYGNNRLAF, and TCRβ with a CDR3 sequence, CASTLGTGGSEQYF, both of which were identified as expanded clonotypes in the SARS-CoV-2 peptide-activated fraction (**Figure 3D**), as well as another clone of CD8T_eff_ (CDR3α: CLVGGGNKLTF, CDR3β: CSVGGGQDNTEAFF). In contrast, among CD8T_eff_ in the patient’s CSF, there was no clonotype reactive to influenza peptides (**Supplementary Figure 7**). Taken together, these two clonotypes were identified as SARS-CoV-2-specific.

The same assay identified no CD4T_eff_ clonotype responsive to SARS-CoV-2 spike peptides (**Supplementary Figure 8**).

SARS-CoV-2-specific T cell clones were localized to a T cell cluster characterized by the high expression of CCL5, HLA-DRB1, HLA-DRA, HLA-DQB1, and GZMK and low expression of cytotoxic genes, such as GNLY, GZMB, and PRF1, in the UMAP plot of scRNA-seq data of the same patient’s PBMCs (**Figures 3E, F**). These results suggest that they are less cytotoxic but potent in driving tissue inflammation. The fraction of the SARS-CoV-2-specific CD8T_eff_ in the peripheral blood decreased after steroid therapy (1.9% to 0.5%, **Supplementary Figure 9**).

### Peripheral NK cells activity and KIR typing

Activity of peripheral NKs also decreased after steroid therapy (**Supplementary Figure 10**). KIR genotyping revealed the presence of KIR2DL1, KIR2DL3, KIR2DL5A, KIR2DL5B, KIR2DS1, KIR2DS4, KIR2DS5, KIR3DL1, KIR3DL2, KIR3DL3, and KIR3DS1, while KIR2DL2, KIR2DS2, and KIR2DS3 were absent.

## Discussion

In this study, we performed single-cell transcriptomic and TCR repertoire analyses of CSF cells and PBMCs to characterize the immune response in a patient who experienced recurrent tumefactive cerebral demyelination after mRNA vaccination. By combining these analyses with a lymphocyte stimulation assay and high-throughput TCR sequencing, we identified SARS-CoV-2-specific CD8T_eff_ clonotypes in the patient. The presence of these clonotypes in the CSF, together with the temporal proximity between vaccination and clinical relapse, raises the possibility that vaccination triggered the recurrence. However, we cannot exclude the possibility that the relapse occurred spontaneously and was only temporally associated with vaccination. How vaccination affects the immune environment of the CNS remains to be elucidated.

Bystander activation is a plausible mechanism of demyelination in this case. SARS-CoV-2-specific clonotypes did not exhibit greater cytotoxicity than other CD8T_eff_ populations but were characterized by a GZMK-high signature and cytokine-producing phenotype. This subset of CD8+ T cells reportedly contributes to the initiation and amplification of inflammation through activating surrounding immune cells (22). Additionally, cell–cell interaction analysis suggested the recruitment and activation of MDCs into the CNS through chemokines and cytokines derived from NK cells and CD8T_eff_. The chemotactic recruitment and activation of antigen-presenting cells within the CNS, together with the release of myelin antigens due to tissue damage, may have facilitated the reactivation of myelin antigen-reactive T cells.

NK cells have recently been recognized as key players in the development of myocarditis after vaccination (23, 24). In our case, the activation of NK cells with increased cytotoxic activity was observed. Our findings raise the possibility that, similar to vaccine-associated myocarditis, the demyelination observed in this case may have been directly mediated by activated cytotoxic NK cells, although the mechanism may not be completely uniform because this patient did not have the high-risk KIR type of myocarditis.

There are some limitations in this report. First, this study is observational, and the direct causal relationship between the vaccine and demyelination is not shown, although it uncovered several important insights regarding the immunological landscape in the CNS after mRNA vaccination. Second, because this study lacked an age-matched control with natural SARS-CoV-2 infection, future studies are needed to determine whether a similar pathological process occurs after SARS-CoV-2 infection or is specific to mRNA vaccination. Third, because only a small number of B cells were recovered from the CSF, this study may have been underpowered to detect differential gene expression between the patient and healthy controls. Therefore, the absence of significant findings in B cells should not be interpreted as evidence against their potential involvement in disease pathogenesis. Finally, due to the limited number of cells in the CSF, we could not analyze CSF cells by scRNA-seq after follow-up. For the same reason, it was not possible to fully analyze the characteristics of SARS-CoV-2-specific CD8+ T cells and their interactions with other cells in the CSF. Further research is needed to clarify the roles of NK cells and CD8+ T cells in this type of post-vaccination demyelinating event.

In conclusion, the observation that vaccine-induced cytotoxic lymphocytes infiltrated the CNS may provide important insights into the immunopathogenesis of post-vaccination demyelination.

## Data Availability

The datasets presented in this study can be found in online repositories. The names of the repository/repositories and accession number(s) can be found below: https://www.ncbi.nlm.nih.gov/geo/, GSE296814.

## References

[1] Mohseni Afshar Z Sharma A Babazadeh A Alizadeh-Khatir A Sio TT Taghizadeh Moghadam MA . A review of the potential neurological adverse events of COVID-19 vaccines. Acta Neurol Belg. (2023) 123:9–44. doi: 10.1007/s13760-022-02137-2 36385246 PMC9668235

[2] Kaulen LD Doubrovinskaia S Mooshage C Jordan B Purrucker J Haubner C . Neurological autoimmune diseases following vaccinations against SARS-CoV-2: a case series. Eur J Neurol. (2021) 29(2):555–563. doi: 10.1111/ene.15147 34668274 PMC8652629

[3] Ozgen Kenangil G Ari BC Guler C Demir MK . Acute disseminated encephalomyelitis-like presentation after an inactivated coronavirus vaccine. Acta Neurol Belg. (2021) 121:1089–91. doi: 10.1007/s13760-021-01699-x 34018145 PMC8136261

[4] Vogrig A Janes F Gigli GL Curcio F Negro ID D'Agostini S . Acute disseminated encephalomyelitis after SARS-CoV-2 vaccination. Clin Neurol Neurosurg. (2021) 208:106839. doi: 10.1016/j.clineuro.2021.106839 34325334 PMC8294707

[5] Patone M Handunnetthi L Saatci D Pan J Katikireddi SV Razvi S . Neurological complications after first dose of COVID-19 vaccines and SARS-CoV-2 infection. Nat Med. (2021) 27:2144–53. doi: 10.1038/s41591-021-01556-7 34697502 PMC8629105

[6] Faksova K Walsh D Jiang Y Griffin J Phillips A Gentile A . COVID-19 vaccines and adverse events of special interest: A multinational Global Vaccine Data Network (GVDN) cohort study of 99 million vaccinated individuals. Vaccine. (2024) 42:2200–11. doi: 10.1016/j.vaccine.2024.01.100 38350768

[7] Stowe J Lopez-Bernal J Andrews N . The risk of acute disseminated encephalomyelitis (ADEM) following covid-19 vaccination in England: A self-controlled case-series analysis. Hum Vaccin Immunother. (2024) 20:2311969. doi: 10.1080/21645515.2024.2311969 38299507 PMC10841003

[8] Morgan HJ Clothier HJ Sepulveda Kattan G Boyd JH Buttery JP . Acute disseminated encephalomyelitis and transverse myelitis following COVID-19 vaccination - A self-controlled case series analysis. Vaccine. (2024) 42:2212–9. doi: 10.1016/j.vaccine.2024.01.099 38350771

[9] Li X Raventós B Roel E Pistillo A Martinez-Hernandez E Delmestri A . Association between covid-19 vaccination, SARS-CoV-2 infection, and risk of immune mediated neurological events: population based cohort and self-controlled case series analysis. BMJ (Clin Res Ed). (2022) 376:e068373. doi: 10.1136/bmj-2021-068373 35296468 PMC8924704

[10] Willison AG Pawlitzki M Lunn MP Willison HJ Hartung H-P Meuth SG . SARS-CoV-2 vaccination and neuroimmunological disease: A review. JAMA Neurol. (2024) 81:179–86. doi: 10.1001/jamaneurol.2023.5208 38227318

[11] Paterson RW Brown RL Benjamin L Nortley R Wiethoff S Bharucha T . The emerging spectrum of COVID-19 neurology: clinical, radiological and laboratory findings. Brain: A J Neurol. (2020) 143:3104–20. doi: 10.1093/brain/awaa240 32637987 PMC7454352

[12] Fekete R Simats A Bíró E Pósfai B Cserép C Schwarcz AD . Microglia dysfunction, neurovascular inflammation and focal neuropathologies are linked to IL-1- and IL-6-related systemic inflammation in COVID-19. Nat Neurosci. (2025) 28:558–76. doi: 10.1038/s41593-025-01871-z 40050441 PMC11893456

[13] Reichard RR Kashani KB Boire NA Constantopoulos E Guo Y Lucchinetti CF . Neuropathology of COVID-19: a spectrum of vascular and acute disseminated encephalomyelitis (ADEM)-like pathology. Acta Neuropathol. (2020) 140:1–6. doi: 10.1007/s00401-020-02166-2 32449057 PMC7245994

[14] Vogrig A Tartaglia S Dentoni M Fabris M Bax F Belluzzo M . Central nervous system immune-related disorders after SARS-CoV-2 vaccination: a multicenter study. Front Immunol. (2024) 15. doi: 10.3389/fimmu.2024.1344184 38375477 PMC10876052

[15] Ishikawa M Shimada Y Ozono T Matsumoto H Ogura H Kihara K . Single-cell RNA-seq analysis identifies distinct myeloid cells in a case with encephalitis temporally associated with COVID-19 vaccination. Front Immunol. (2023) 14. doi: 10.3389/fimmu.2023.998233 36911677 PMC9996085

[16] Gate D Saligrama N Leventhal O Yang AC Unger MS Middeldorp J . Clonally expanded CD8 T cells patrol the cerebrospinal fluid in Alzheimer’s disease. Nature. (2020) 577:399–404. doi: 10.1002/alz.044082 31915375 PMC7445078

[17] Hao Y Hao S Andersen-Nissen E Mauck WM III Zheng S . Integrated analysis of multimodal single-cell data. Cell. (2021) 184:3573–87.e29. doi: 10.1016/j.cell.2021.04.048 34062119 PMC8238499

[18] Ianevski A Giri AK Aittokallio T . Fully-automated and ultra-fast cell-type identification using specific marker combinations from single-cell transcriptomic data. Nat Commun. (2022) 13:1246. doi: 10.1038/s41467-022-28803-w 35273156 PMC8913782

[19] Yu G Wang LG Han Y He QY . clusterProfiler: an R package for comparing biological themes among gene clusters. Omics. (2012) 16:284–7. doi: 10.1089/omi.2011.0118 22455463 PMC3339379

[20] Efremova M Vento-Tormo M Teichmann SA Vento-Tormo R . CellPhoneDB: inferring cell–cell communication from combined expression of multi-subunit ligand–receptor complexes. Nat Protoc. (2020) 15:1484–506. doi: 10.1038/s41596-020-0292-x 32103204

[21] Bolotin DA Poslavsky S Mitrophanov I Shugay M Mamedov IZ Putintseva EV . MiXCR: software for comprehensive adaptive immunity profiling. Nat Methods. (2015) 12:380–1. doi: 10.1038/nmeth.3364 25924071

[22] Jonsson AH Zhang F Dunlap G Gomez-Rivas E Watts GFM Faust HJ . Granzyme K(+) CD8 T cells form a core population in inflamed human tissue. Sci Transl Med. (2022) 14:eabo0686. doi: 10.1126/scitranslmed.abo0686 35704599 PMC9972878

[23] Barmada A Klein J Ramaswamy A Brodsky NN Jaycox JR Sheikha H . Cytokinopathy with aberrant cytotoxic lymphocytes and profibrotic myeloid response in SARS-CoV-2 mRNA vaccine–associated myocarditis. Sci Immunol. (2023) 8:eadh3455. doi: 10.1126/sciimmunol.adh3455 37146127 PMC10468758

[24] Tsang HW Kwan MYW Chua GT Tsao SSL Wong JSC Tung KTS . The central role of natural killer cells in mediating acute myocarditis after mRNA COVID-19 vaccination. Med. (2024) 5:335–47.e3. doi: 10.1016/j.medj.2024.02.008 38521068

