# Supplementary material for: Identification of SARS-CoV-2-specific cytotoxic lymphocytes in cerebrospinal fluid of a patient with cerebral demyelination Post mRNA vaccination
Source: Front Immunol. 2026 Sep 21;17:1920061. doi: 10.3389/fimmu.2026.1920061 (PMC13634803; doi:10.3389/fimmu.2026.1920061)
Supplement: Supplementary file 1 [file DataSheet1.pdf]

**Supplemental Table S1. Laboratory test results**	
Complete blood count	
Leukocytes, /μL	6,700
Erythrocytes, 10^4//μL	476
Hematocrit, %	41.5
Hemoglobin, g/dL	14.4
Platelets, 10^3//μL	243
Neutrophils, %	52.3
Lymphocytes, %	30.7
Eosinophils, %	9.4
Monocytes, %	7.2
Basophils, %	0.4
Blood chemistry	
Urea nitrogen, mg/dL	14
Creatinine, mg/dL	0.88
Sodium, mEq/L	143
Potassium, mEq/L	3.5
Chloride, mEq/L	107
C-reactive protein, mg/dL	0.50
Serum antibodies	
Aquaporin-4	−
Myelin oligodendrocyte glycoprotein	−
Serum anti-neuronal antibodies	
Amphiphysin	−
CV2	−
PNMA2 (Ma2/Ta)	−
Ri	−
Yo	−
Hu	−
Recoverin	−
SOX1	−
Titin	−
Zic4	−
GAD65	−
Tr (DNER)	−
Cerebrospinal fluid examinations	
Oligoclonal bands	−
IgG index	0.60

Supplemental Table S2. Number of cells according to the cell types

	Patient	Control
Naive CD4+ T cells	54	1972
Effector CD4+ T cells	138	4759
Effector CD8+ T cells	145	2397
Natural killer cells	56	193
B cells	11	28
Monocytes	40	113
Myeloid Dendritic cells	50	250
Plasmacytoid Dendritic cells	1	44

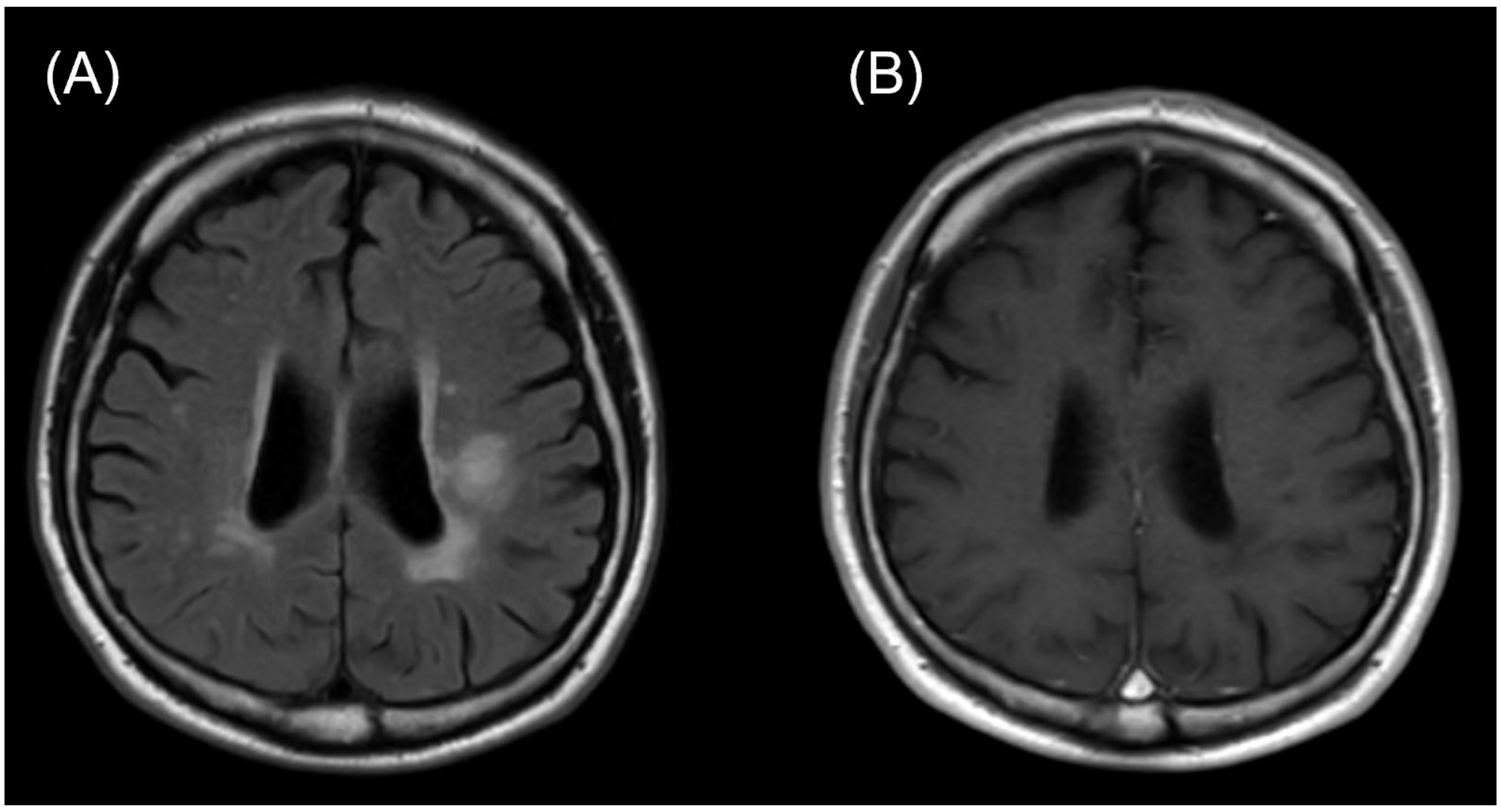

Supplemental Figure S1.

Magnetic resonance images 7 months after the onset. A, T2 FLAIR and B, contrast-enhanced T1.

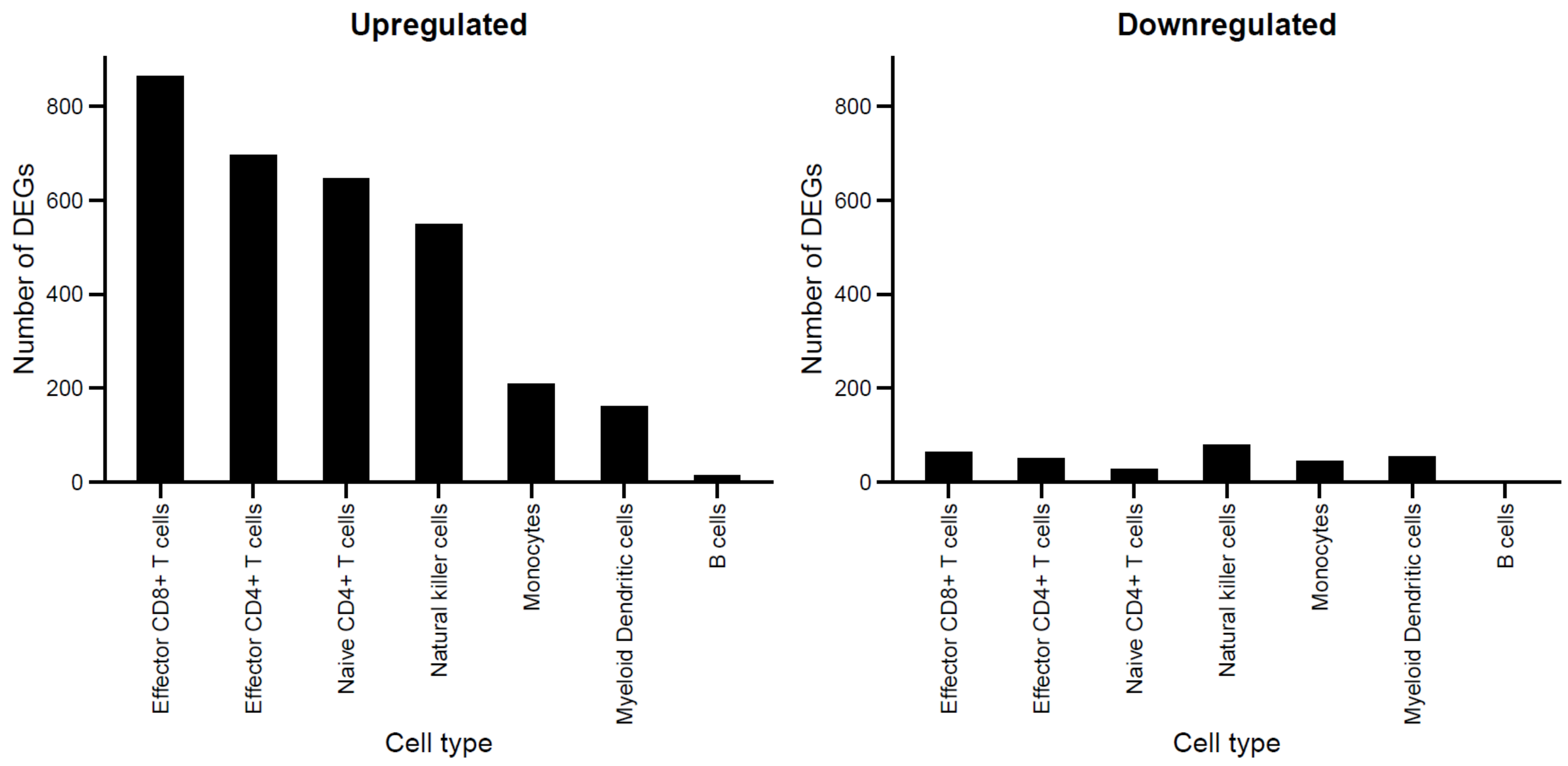

Supplemental Figure S2.

Numbers of differentially expressed genes across cerebrospinal fluid immune cell types. Bar plots show the numbers of significantly upregulated and downregulated genes in patients compared with healthy controls for each cell type.

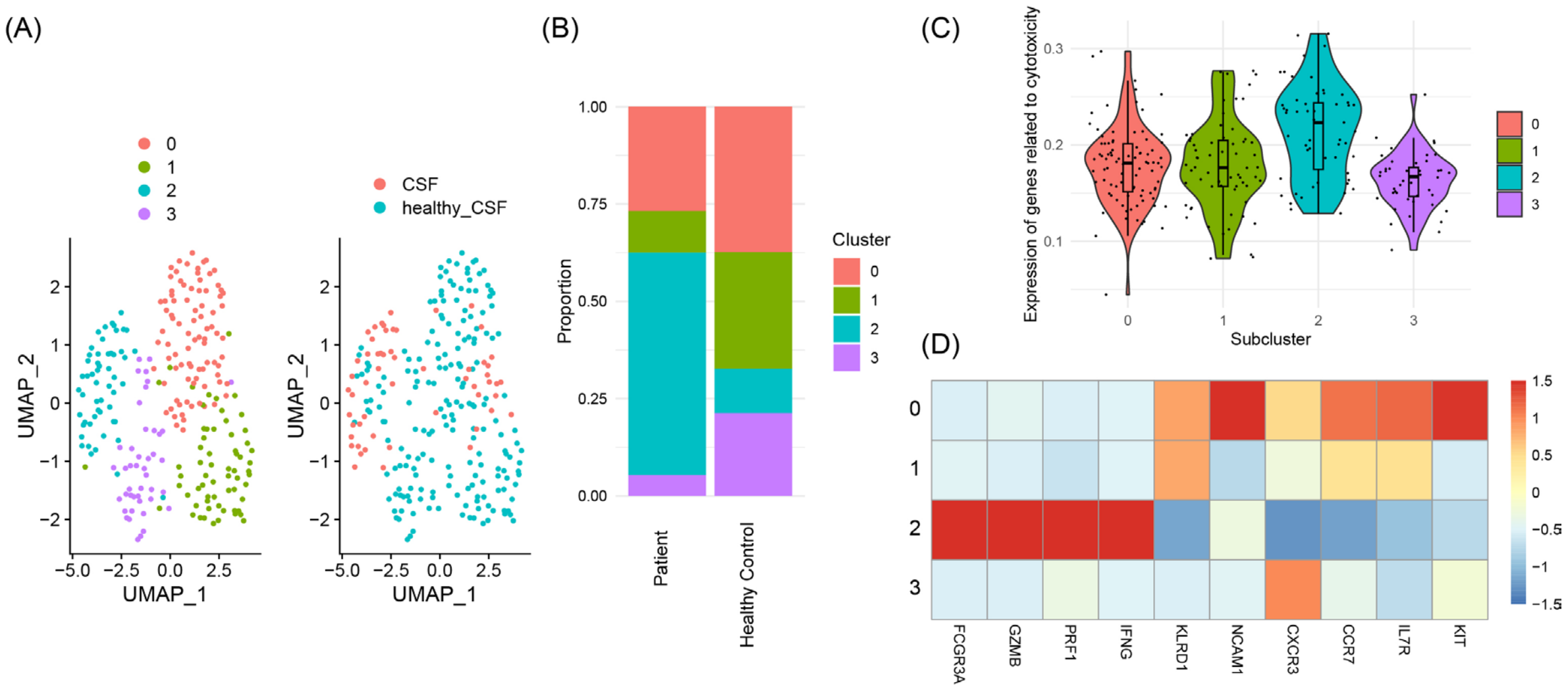

Supplemental Figure S3.

Re-clustering of NKs in CSF. **(A)** UMAP plot of the result of re-clustering. (B) Proportion of each cluster according to the sample. (C) Expression of gene related to cytotoxicity. (D) Expression of marker genes of mature and immature NKs.

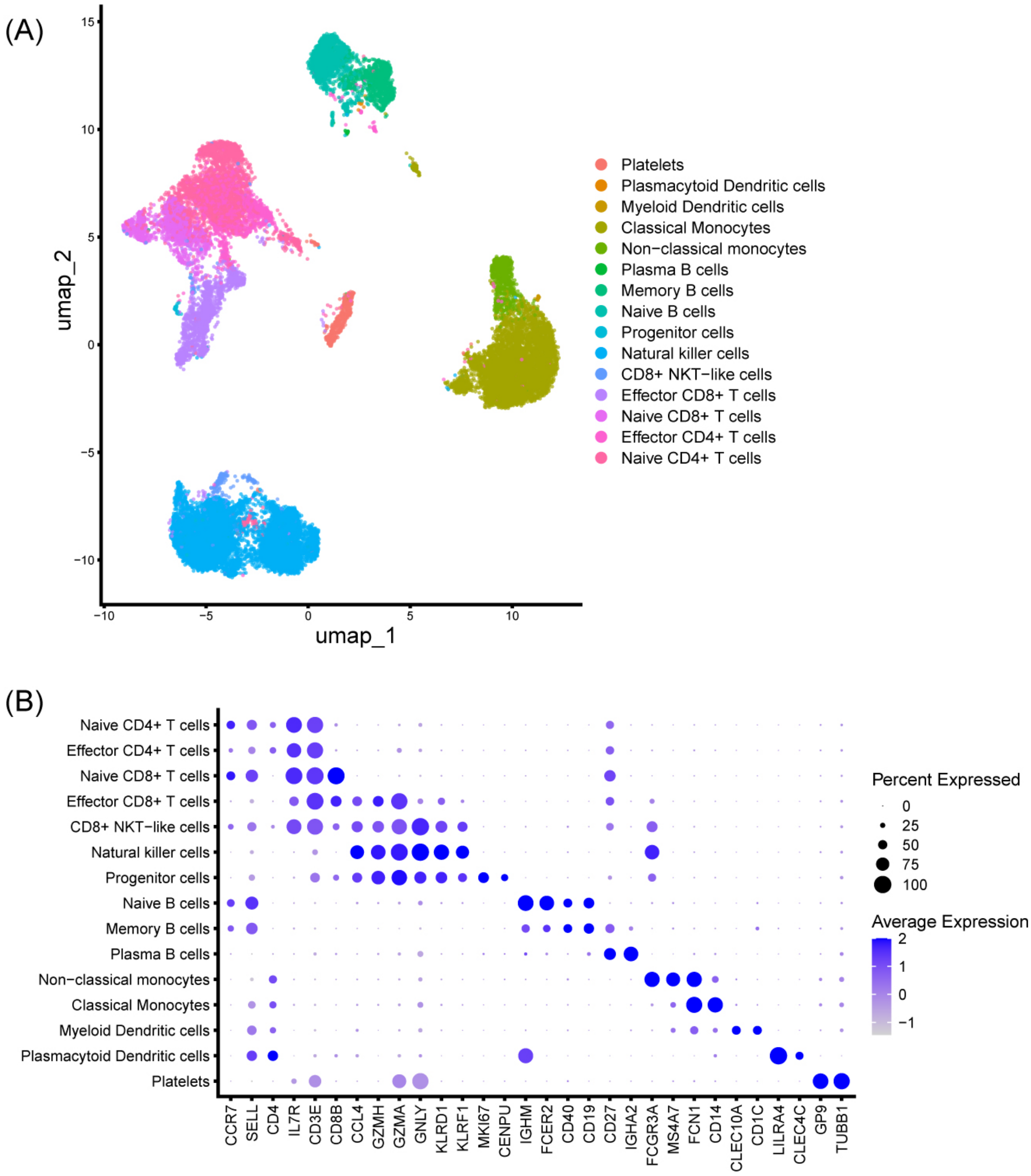

Supplemental Figure S4.

Identifying cell type of PBMCs. (A) UMAP plot for the PBMCs (pre-treatment 12,014; after steroid therapy: 8,207; after recovery: 3,719). Cell type was automatically annotated using ScType. (B) Dot plot for the marker gene expression.

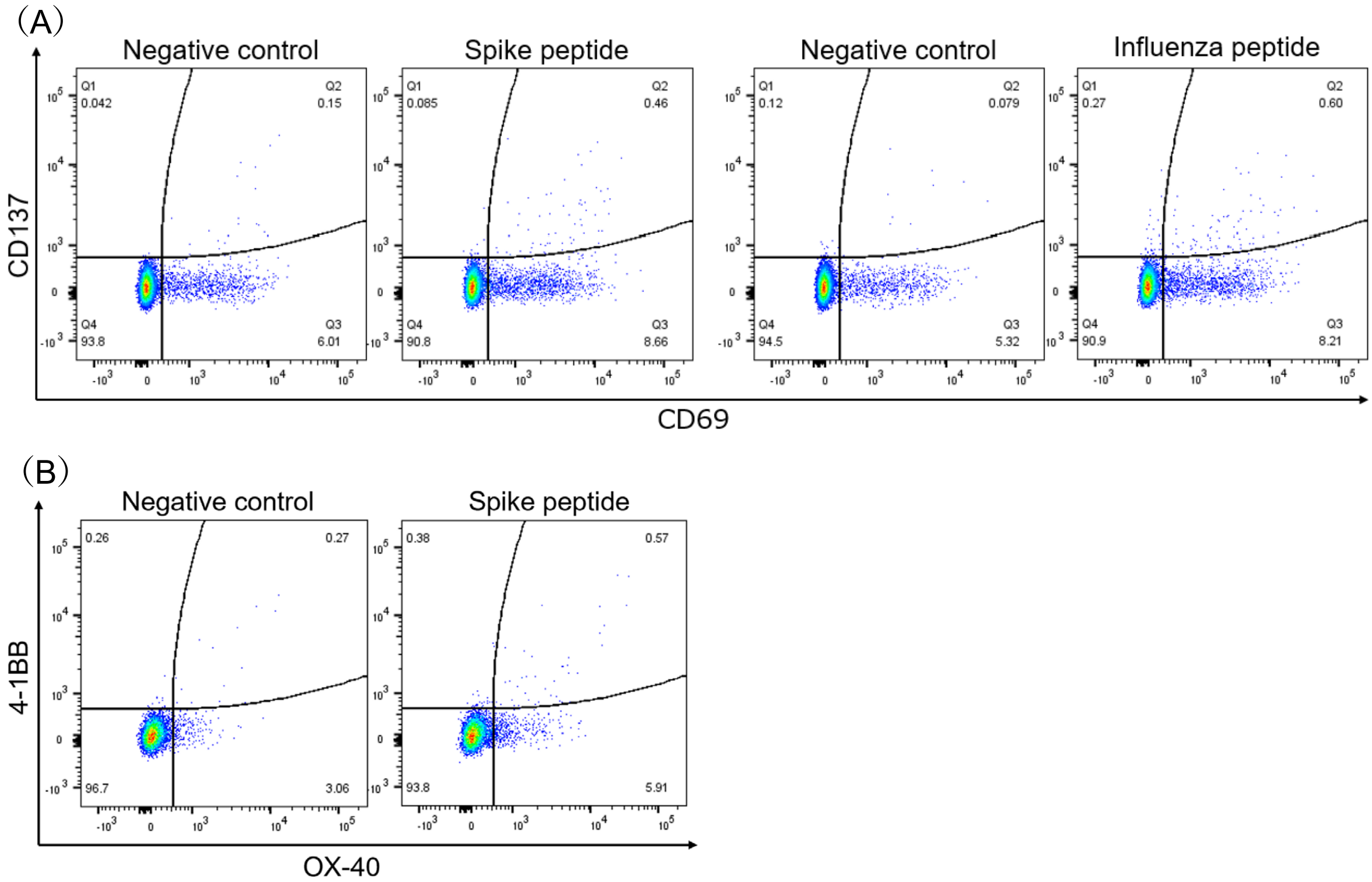

Supplemental Figure S5.

Representative flow cytometric plots of **(A)** CD8+ T cells and **(B)** CD4+ T cells. Double positive ratios are shown for SARS-CoV2 spike peptide stimulation, influenza peptide stimulation, and each negative control.

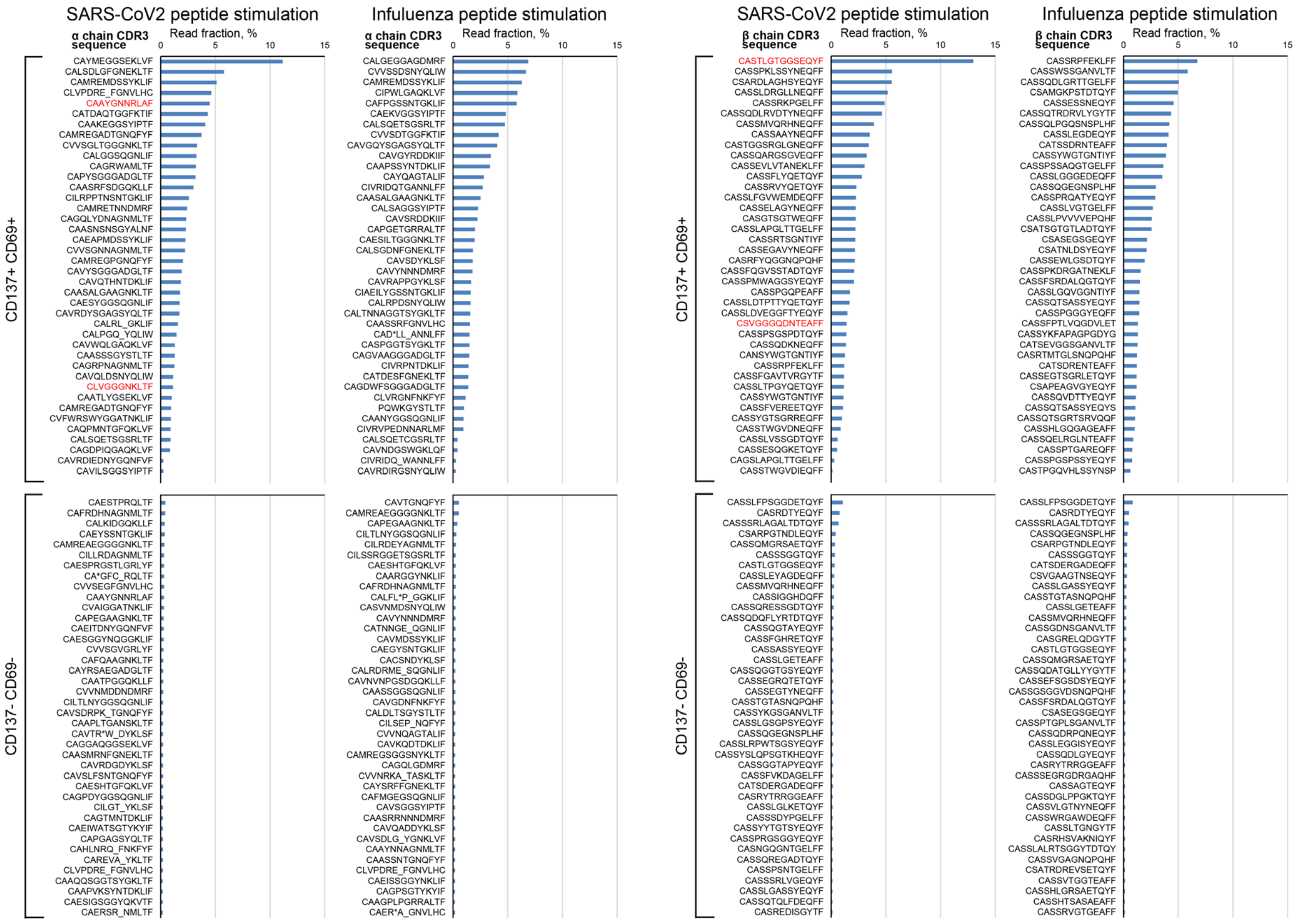

Supplemental Figure S6.

Bulk TCR sequencing of peripheral CD8+ T cells was performed individually according to the stimuli and the response. The percentage of the read of each CDR3 sequence to the entire read count of the group were calculated as read fraction. The top 40 CDR3 sequence of α and β chain are listed. After both stimulations, expansion of some CDR3 sequences were observed. The CDR3 sequences which were also identified as a clonotype in the patient's CSF by single cell TCR sequencing are highlighted in red.

CDR3 amino acid sequences		SARS-CoV2 peptide stimulation		Influenza peptide stimulation	
α chain	β chain	α chain	β chain	α chain	β chain
CALSDHNYGQNFVF	CASSQGEGNSPLHF	ND	0.00	0	0.89
CAAYGNNRLAF	CASTLGTGGSEQYF	0.97	0.98	0.01	0.00
CAMREAYSGAGSYQLTF	CASSFLSGANVLTF	ND	ND	ND	ND
CAAPLNYGGQNFVF	CASSLTANQPQHF	ND	ND	ND	ND
CALRGKAAGNKLTF	CSVEWEHETQYF	ND	ND	ND	ND
CAVGYNAGNMLTF	CASSLGLKETQYF	ND	ND	ND	0
CAVRDLYSGGYQKVTF	CASSRSGSEQYF	ND	ND	ND	0
CAESYGQNFVF	CASSLVGLKVYGYTF	0	ND	ND	ND
CAGFTTSGTYKYIF	CASSELAGGPNEQFF	ND	ND	ND	ND
CAMKYTGNQFYF	CASSIRGVTYQPQHF	ND	ND	ND	ND
CAVAGGTSYGKLTF	CASSIGGHDQFF	0	ND	ND	0
CAVRSYNTDKLIF	CASTFDTGSIYTGELFF	0	ND	0	0
CAVSDRDNARLMF	CSAGRTSGPTDTQYF	ND	ND	ND	ND
CAVSYTNAGKSTF	CASSLTGTGTPLMNTEAFF	ND	ND	ND	0
CAVTQGSSNTGKLIF	CASSALGWGDEQFF	ND	ND	ND	ND
CLVGGGNKLTF	CSVGGGQDNTEAFF	0.99	0.97	0.67	ND
CVVSEDNARLMF	CASELPASSIGKLFF	0	ND	ND	ND
NA	CASSVLGTNYNEQFF	ND	ND	ND	0

Supplemental Figure S7.

The CDR3 amino acid sequence of the expanded effector CD8+ T cell clonotype identified in CSF by single cell TCR sequencing are presented in the same row. Fraction of the read counts detected in CD69+CD137+ cells by bulk TCR sequencing to the entire (CD69+CD137+ and CD69-CD137- cells) read counts of the sequence are shown according to the stimulation. SARS-CoV2 spike peptide or influenzae peptide was used as the stimulation. The sequences are highlighted in red when both α and β chain of the clonotype are responsive to the stimulus. ND stands for that the sequence was not detected in the bulk TCR sequencing of the PBMCs although it was detected by single cell TCR sequencing of the CSF cells. NA for not assigned.

CDR3 amino acid sequences		SARS-CoV2 peptide stimulation	
α chain	β chain	α chain	β chain
CIVSWSPDNQGGKLIF	CASAEGSNYGYTF	ND	ND
CVVSAETGNQFYF	CASSLYVGYNQPQHF	ND	ND
CAASNLGRAGSYQLTF	CASSSRAVQRGTQYF	ND	ND
CAGGGNQFYF	CSAENGEAFF	ND	ND
CATAAGGTSYGKLTF	CSVPKGQGDEAFF	ND	ND
CATDANDGQKLLF	CASSSGQEQFF	ND	ND
CAVNGEAGNTPLVF	CASRDGWSANTIYF	ND	ND
CAVRTPTGGGNKLTF	CASSQITEGTQYF	ND	ND
CAVVDTGTASKLTF	CASSQAGVPEAFF	ND	0
CLVVGAAGNKLTF	CASSLGGPVDEQYF	ND	ND
CAAPKGSGGSNYKLTF	CASSLWSLDRGTEAFF	ND	ND
CAASDQAGTALIF	CASSLPLAGGGTGELFF	ND	ND
CAASFNAGGTSYGKLTF	CSASPGTRSYGEQYF	ND	ND
CAASIREDSWGKLQF	CSASSGNTIYF	ND	ND
CAASLPGYALNF	CASSFQGTGDGYTF	ND	ND
CAASPNYGGATNKLIF	CSASPPGSNSQPQHF	0	0
CAASSDDSWGKLQF	CSAPGSYQTQYF	ND	ND
CAAWRGNKLVF	CASAVISGGGETQYF	ND	ND
CAEIKNAGNMLTF	CSAVASGAGGTDTQYF	ND	ND
CAENILNSGYALNF	NA	ND	ND
CAENSGGGADGLTF	CSARDPTSGPTDTQYF	ND	ND
CAESDTNAGKSTF	CASIGQGHEQFF	ND	ND
CAESSLEGKLIF	CASSLAGVDTEAFF	ND	ND
CAETGLTGGGNKLTF	CSAREGSGELFF	ND	ND
CAETGMDSSYKLIF	CASGSDRRYNEQFF	ND	ND
CAETTTGANNLFF	CASSFSPGLAGITDTQYF	ND	ND
CAFLETSGSRLTF	CASSWGGTHEQYF	ND	ND
CAGDGDSGAGSYQLTF	CASSQEGGLAGGYNEQFF	ND	ND
CAGHNQGGKLIF	CASSQDDRGASEQYF	ND	0.00
CAGHSNDYKLSF	CASSSNYEQYF	ND	ND
CAGRDNYGQNFVF	CSASTGVREQYF	ND	ND
CAGRRFGNEKLTF	CASSVDGVATEAFF	ND	ND
CAGSSNTGKLIF	CASSLTGPLHF	ND	ND
CAGYNAGNMLTF	CASTRRGAGNEQFF	ND	ND
CAITTNAGKSTF	CASSLELDGGFSTGELFF	ND	ND
CALDPTYNQGGKLIF	CASSLGTVIPYGYTF	ND	ND
CALKIDGQKLLF	CASGRELQDGYTF	ND	ND
CALNGKLTF	CASSLAGVNTGELFF	ND	0
CALRARSSGYALNF	CASSPQTGWYEQYF	ND	ND
CALRGKAAGNKLTF	CSVEWEHETQYF	0	0
CALSDNNARLMF	CASSLMYQDSPLHF	0	ND
CALSEARLYNQGGKLIF	CASSVDRGFYEQYF	ND	ND
CALSVGAKGFGNVLHC	CASSLGQGWTDTQYF	ND	ND
CALSVITQGGSEKLVF	CASSWDREGETQYF	ND	ND
CAMMRGAGSYQLTF	CASSEGWLAGAEQYF	ND	ND
CAMNTGGQLTF	CASSLEPGGGSYF	ND	ND
CAMREAYSGAGSYQLTF	CASSQHGGSNEQFF	ND	ND
CAMRELSWGGNTPLVF	CAWSGGGYGDTEAFF	ND	ND
CAMREPLTTDKLIF	ASSGPGQGAVPGTYNEQFF	ND	ND
CAMRGYGNTGFQKLVF	CASSSSAYEQYF	ND	ND
CAMRVGNTGGFKTIF	CASSHPSGNNSPLHF	ND	ND
CAMSAGNKLVF	CASSLGQTSGGGETQYF	ND	ND
CAMSSGGTSYGKLTF	CAASPGSDGQKLLF	0	ND
CAMSVAGANNLFF	CASSQSGTSGGFSLNEQFF	ND	ND
CAMSYDNYGQNFVF	CASTPRELAGYNEQFF	ND	ND
CAPATQGGSEKLVF	CASSLPGTGRSGELFF	ND	ND
CAPLARVRRDDKIIF	CASSQAPGGETEAFF	ND	ND
CASDTGRRALTF	CSARLTSKMVGEQFF	ND	ND
CASQGGSQGNLIF	CSAQRGYQGFGDEQFF	ND	ND
CASTDVEYGNKLVF	CASSLIWGYEQYF	ND	ND
CASVNTNAGKSTF	CASSLGTGGLNYGYTF	ND	ND
CASYSGGGADGLTF	CASSPRPQPQHF	0	ND
CATDDASGSRLTF	CATSDPDYFNTGELFF	ND	ND
CATDDNDYKLSF	CASSLGGTGPNQPQHF	ND	ND
CATDESGAGSYQLTF	CAWSGWESYNSPLHF	ND	ND
CATDGSTGTASKLTF	CASRAYNEQFF	ND	ND
CATDPYSGGSNYKLTF	CASSQGNEQFF	ND	ND
CATGMFSGGYNKLIF	CSAHGQGAARDEKLFF	ND	ND
CAVCDMNRDDKIIF	CASRGMSVGNEQFF	ND	ND
CAVEASGAGSYQLTF	CSVKGQGMRLFF	ND	ND
CAVEEPWGSQGNLIF	CASSGIHRGSGLNEQFF	ND	ND
CAVGITSGTYKYIF	CASSQNIGNSGNTIYF	ND	ND
CAVGVGGYQKVTF	CASTDYNEQFF	ND	ND
CAVISNFGNEKLTF	CSARWGGSSYEQYF	0	ND
CAVISSRLTF	CASSLLQQNSPLHF	ND	ND
CAVLLSGSARQLTF	CASSPSGQSTDTQYF	ND	ND
CAVNGAASGYGNKLVF	CASSQDWALQETQYF	ND	ND
CAVNKDNNARLMF	CSADPGGNGNQPQHF	ND	ND
CAVNPWGGGGADGLTF	CASSLVQGSYEQYF	ND	ND
CAVPILRGYNFNKFYF	CASSIGKEVEKLFF	ND	ND
CAVQGNSGYALNF	CASSPMEGTGNTIYF	ND	ND
CAVRDGRFGNVLHC	NA	ND	ND
CAVRDRSISNFGNEKLTF	CASSARGFFYGYTF	ND	ND
CAVRGDPIDNARLMF	CSAEDPDGTAFYEQYF	ND	ND
CAVRNIAGGTSYGKLTF	CASSQDRTRGGELFF	0	0
CAVRTDKLIF	CSPEPATGRLFSPLHF	0	0
CAVRVDNFGNEKLTF	CSAHRDSQETQYF	ND	ND
CAVSEKSGKLTF	CASSSGRGAEETQYF	ND	ND
CAVSKEGYQKVTF	CASRKLTGTGVEKLFF	ND	ND
CAVVGLAGAQKLVF	CASSWAAGQTQYF	0	0
CAVWNTGNQFYF	CASGGGKDTQYF	ND	ND
CAYAEGKLIF	CASSFPDGSFGGYTF	ND	ND
CAYPENRDDKIIF	CASSGGGQPYGYTF	0.44	0.28
CAYSREYGGATNKLIF	CASSFTGTALGVEQYF	0	0
CGADPRGGAQKLVF	CASRGNTGELFF	ND	ND
CIVGQGAQKLVF	CSVRDRGFGEQYF	ND	ND
CIVRAGAVKAAGNKLTF	CSVEGEGGLAFF	ND	ND
CIVRAPIDDKIIF	CASSLGTAYNEQFF	0.13	ND
CIVRSYSTSGTYKYIF	CASRAGTGLSYEQYF	ND	ND
CIVRVEGGSQGNLIF	CASTPGLGRGSYNEQFF	ND	0
CLTVRGSGGSNYKLTF	CASSPQYLHGLNYGYTF	ND	ND
CLVGDGNNQGGKLIF	CSAKTGTSGYNEQFF	ND	ND
CLVGVDSNYQLIW	CAWSGGLAGLTDTQYF	ND	ND
CLVGYNTNAGKSTF	CASSLTPGPNEKLFF	ND	ND
CVVNSNSGYALNF	CASSIYRDRWYQETQYF	ND	ND
NA	CASRAGTGLSYEQYF	ND	ND
NA	CASSHLQGANQPQHF	ND	ND
NA	CASSLQTGLYEQYF	ND	ND
NA	CASSLRGAPNSPLHF	ND	ND
NA	CASSPPYSTSSGELFF	ND	ND
NA	CASSSGQEQFF	ND	ND
NA	CASSTTADTEAFF	ND	ND
NA	CASSVDGVATEAFF	ND	ND
NA	CASSVDRLDNEQFF	ND	ND
NA	CASSVLGTNYNEQFF	ND	ND
NA	CSAKTGTSGYNEQFF	ND	ND

Supplemental Figure S8.

We separated CD4+ T cells from the stimulated PBMCs. After the stimulation, 0.57% of the peripheral CD4+ T cells responded (OX-40+ and 4-1BB+) to SARS-CoV-2, whereas 0.27% reacted to vehicle alone. The CDR3 amino acid sequence of the effector CD4+ T cell clonotype identified in CSF by single cell TCR sequencing are presented in the same row. Fraction of the read counts detected in OX-40+ and 4-1BB+ cells by bulk TCR sequencing to the entire read counts of the sequence are shown according to the stimulation. SARS-CoV2 spike peptide was used as the stimulation. No clonotype responsive to the stimulus was detected. ND stands for that the sequence was not detected in the bulk TCR sequencing of the PBMCs although it was detected by single cell TCR sequencing of the CSF cells. NA for not assigned.

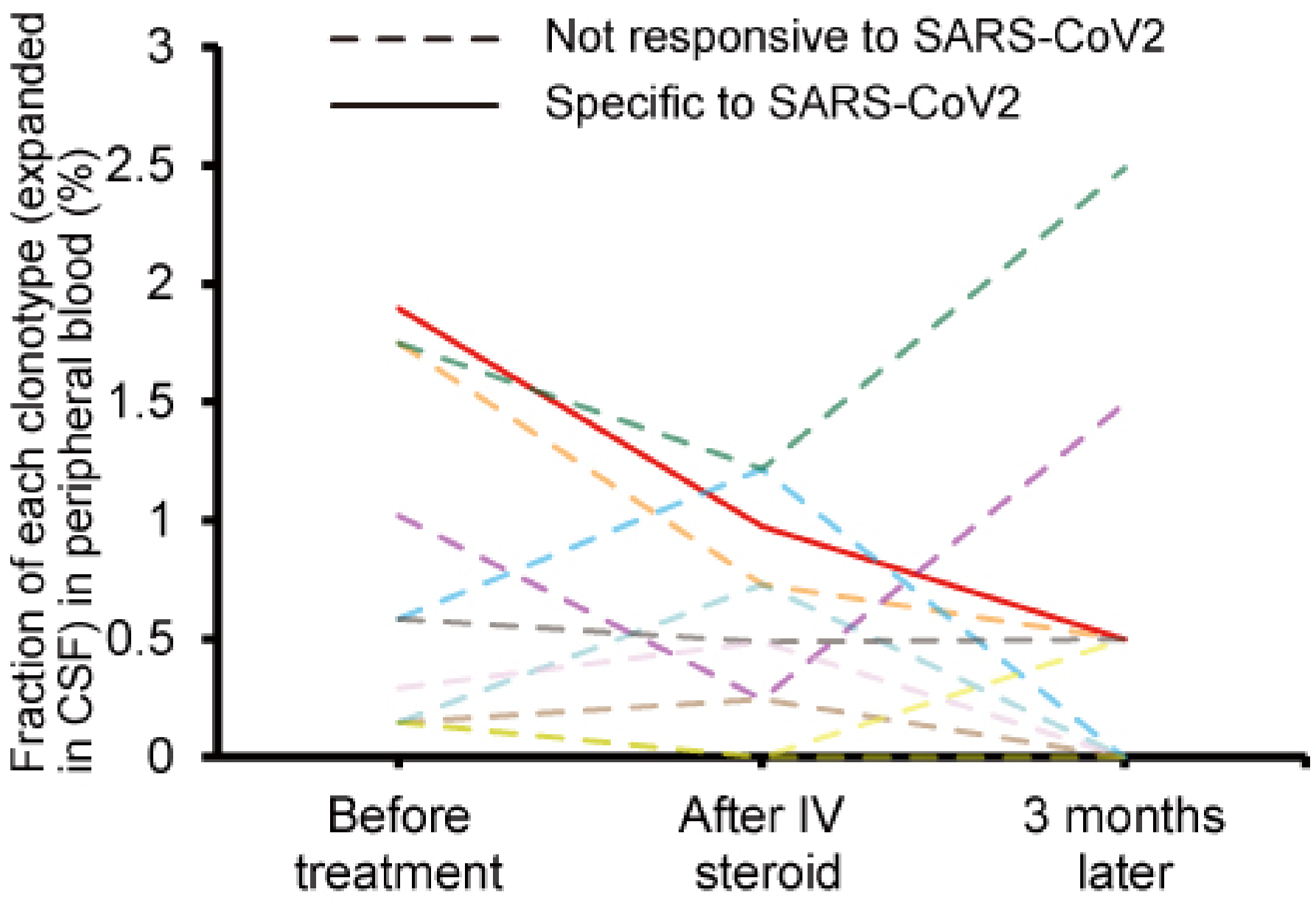

Supplemental Figure S9.

Temporal change of the proportion of the clonotypes among the peripheral $CD8T_{eff}$. Only clonotypes which were expanded in the CSF are shown. Each color represents a clonotype. The clonotype specific to SARS-CoV-2 decreased after the treatment (highlighted in red).

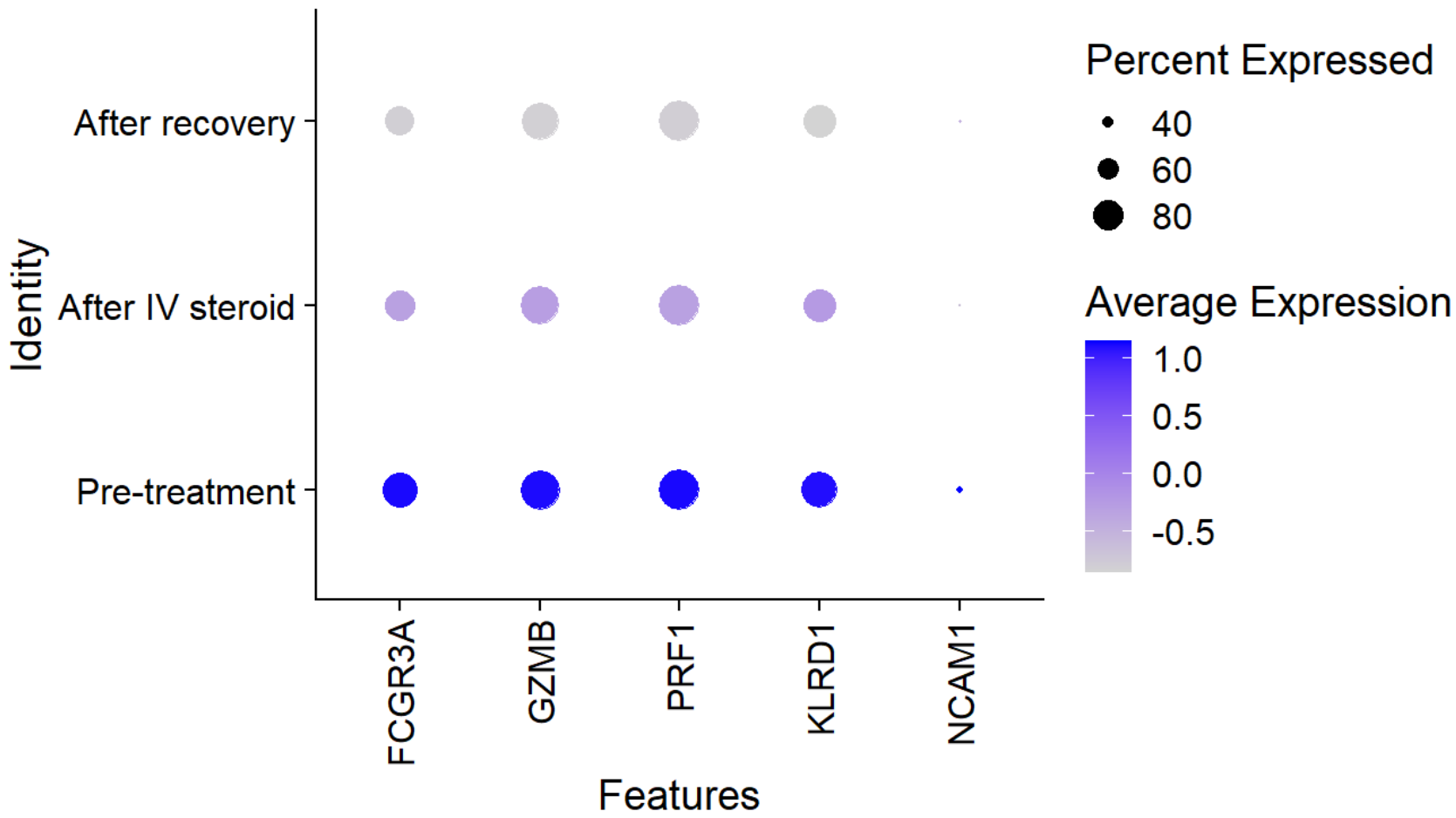

Supplemental Figure S10.

Temporal change of the canonical marker gene expressions by peripheral NKs.
